# Determination of Dairy Cattle Euthanasia Criteria and Analysis of Barriers to Humane Euthanasia in the United States: The Veterinarian Perspective

**DOI:** 10.3390/ani10061051

**Published:** 2020-06-18

**Authors:** Brooklyn K. Wagner, Mary Caitlin Cramer, Heather N. Fowler, Hannah L. Varnell, Alia M. Dietsch, Kathryn L. Proudfoot, Jan Shearer, Maria Correa, Monique D. Pairis-Garcia

**Affiliations:** 1 Department of Population Health and Pathobiology, College of Veterinary Medicine, North Carolina State University, Raleigh, NC 27606, USA; bwagner2@ncsu.edu (B.K.W.); hlvarnel@ncsu.edu (H.L.V.); correa@ncsu.edu (M.C.); 2 Department of Animal Science, College of Agricultural Sciences, Colorado State University, Fort Collins, CO 80523, USA; catie.cramer@colostate.edu; 3 College of Veterinary Medicine, Iowa State University, Ames, IA 50011, USA; hnfowler@gmail.com; 4 School of Environment and Natural Resources, College of Food, Agricultural, and Environmental Sciences, The Ohio State University, Columbus, OH 43210, USA; dietsch.29@osu.edu; 5 Departments of Health Management and Companion Animals, Atlantic Veterinary College, University of Prince Edward Island, Charlottetown, PE C1A 4P3, Canada; kproudfoot@upei.ca; 6 Department of Veterinary Diagnostic and Production Animal Medicine, College of Veterinary Medicine, Iowa State University, Ames, IA 50011, USA; jks@iastate.edu

**Keywords:** animal welfare, survey, cow, calf, heifer, euthanasia

## Abstract

**Simple Summary:**

Making and carrying out euthanasia decisions on dairy farms is complex and decisions are often challenging and multi-factorial. Training caretakers to be confident in their abilities to make timely decisions about euthanasia is critical. Veterinarians are often asked to serve in the role of training caretakers on euthanasia. Given the valuable role veterinarians play regarding dairy cattle euthanasia, the objective of this two-part study was to evaluate the main factors influencing euthanasia practices on dairy farms across the United States (US) by recruiting experienced dairy cattle veterinarians to participate in either an online survey (Part I) or in-person focus groups (Part II). Survey results indicated variability in health condition management amongst dairy cattle veterinarians, and only 60% of serviced facilities have a written euthanasia protocol in place. Three main themes of euthanasia decision-making were identified across focus group discussions and included logistical, animal, and human Factors. In addition, participants frequently expressed frustration with protocol limitations and requirements. These results suggest the need for the development and implementation of science-based standards to improve cattle welfare on-farm by promoting consistency in euthanasia decision-making across the US dairy industry.

**Abstract:**

When dairy cattle become ill or injured to the extent that recovery is unlikely or impossible, on-farm euthanasia should be used as a tool to eliminate pain and suffering. Our study aimed to identify decision-making criteria and the most common factors considered by veterinarians when making and carrying out euthanasia decisions. Dairy cattle veterinarians were recruited to participate in an online survey (Part I, 61 surveys collected) or in one of three focus groups (Part II, 4–10 veterinarians/group, *n* = 22). Part I (survey): Surveyed veterinarians varied regarding health condition management and demonstrated a strong proclivity to treat compromised cattle, mirroring trends amongst dairy producers identified in previous research. Sixty percent of respondents indicated that most facilities for which they serve as the primary veterinarian have a written euthanasia protocol in place. Part II (focus groups): Three main themes about euthanasia decision-making (logistical, animal, and human) were identified from focus group discussions. Discussions focused primarily on logistical factors such as financial considerations and client/public perceptions. Development of specific standards for euthanasia, alongside interactive training programs for dairy veterinarians and producers are vital next steps to improving cattle welfare and consistency in euthanasia decision-making across the United States dairy industry.

## 1. Introduction

Veterinarians serve as experts in animal husbandry and disease for livestock producers and play a critical role in training caretakers. In the United States (US) dairy industry, veterinarians are often responsible for training caretakers on euthanasia and assist on-farm with developing euthanasia protocols to ensure timeliness to minimize animal suffering [1]. However, timely euthanasia remains a challenge for dairy farms. In 2020, a Canadian study indicated that 48% of reported mortality events in adult cows were classified as ‘unassisted deaths’ [2]. Furthermore, more than 20 undercover videos have been taken in commercial dairy facilities throughout the US since 1999 and have captured images and videos of compromised cattle that required euthanasia but instead remained on the farm for undisclosed lengths of time.

Some basic euthanasia guidelines are provided by industry organizations such as the American Veterinary Medical Association (AVMA; Schaumburg, IL, USA) [3], American Association of Bovine Practitioners (AABP) [4], and National Milk Producer’s Federation Farmers Assuring Responsible Management Program (NMPF FARM™; Arlington, VA, USA) [5]. However, to date, the industry has not established nationally recognized standards for specific conditions in dairy cattle that warrant euthanasia nor identified specific timelines for each condition.

A lack of clear standards for timely euthanasia has resulted in inconsistencies amongst US dairy veterinarians in regard to implementing euthanasia on-farm [1]. To ensure good animal welfare on dairy farms, a better understanding of the challenges and barriers to implementing timely euthanasia is needed. Given the valuable role the veterinarian serves specific to on-farm euthanasia, the objective of the present study aimed to identify quantitative and qualitative decision-making criteria for on-farm euthanasia of dairy cattle amongst veterinarians using online surveys and in-person focus groups.

## 2. Materials and Methods

All research was reviewed and approved by The Ohio State University IRB Committee for Human Subjects Research.

### 2.1. Part I: Survey

#### 2.1.1. Development and Participant Recruitment

An internet survey was created utilizing methods described by Wagner et al. in 2020. Briefly, Qualtrics^®^ software (v. January, 2019; Provo, UT, USA) was used as a platform for surveys, and content was developed and validated by authors with expertise in euthanasia, cattle health, and survey methodology [1].

Dairy cattle veterinarians were targeted using the American Association of Bovine Practitioners (AABP) listserv. Members who were involved in euthanasia decision-making for dairy cattle were asked to participate in the survey. Members were sent an initial email that included a link to the online survey. To follow-up and encourage maximum participation, three additional recruitment emails were sent. In total, the survey was available for six weeks (7 January–15 February, 2019). The number of AABP members who received emails via listserv at the time of survey distribution was unknown; therefore, response rate could not be reliably calculated.

#### 2.1.2. The Survey

The survey collected demographic information including age, gender, years of experience working with cattle as a veterinarian and the number of facilities servicing as the primary veterinarian. In addition, respondents were asked to provide information regarding serviced farms and euthanasia procedures. A full list of survey questions can be found in Appendix A.

Respondents were then asked to make decisions regarding the management of 15 designated health conditions in accordance with the methodology and format described by Wagner et al. [1].

Survey data were exported, and completed surveys were coded manually by one researcher to support coding consistency and control for inter-individual bias. Respondents were able to complete as much or as little of the survey as they wanted, in accordance with IRB protocol.

#### 2.1.3. Data Analysis

Data were exported in spreadsheet format and checked for data entry and coding errors. Any “non-responses” were set as a missing value. Descriptive statistics were obtained for all survey variables, including frequency for nominal or ordinal data, and mean, standard deviation, median, and range for continuous variables. Data distributions for numerical variables were checked to determine if there were data gaps and variable categorizations were needed to define new variables. The T-tests, or contingency-table tests, were used to determine bivariate associations with the outcome of interest with a pretest probability value set at α ≤ 0.05.

### 2.2. Part II: Focus Group

#### 2.2.1. Development and Participant Recruitment

Dairy cattle veterinarians were targeted via two dairy veterinarian conferences held in the US (American Association of Bovine Practitioners Annual Conference 2019, St. Louis, MO, USA; Ohio Dairy Veterinarians Meeting 2019, Columbus, OH, USA). With support from conference organizers, a recruitment email was sent to registered participants. In order to participate in a focus group, participants were required to have experience as a dairy cattle veterinarian and to be familiar with the usual euthanasia practices and related training provided on the farm. Interested individuals contacted the research team and focus groups were arranged during each of the conferences (two held at AABP Annual Conference and one held at ODV Meeting). Focus group participation was optional and voluntary.

#### 2.2.2. Focus Group Format

A total of three focus groups were conducted as described by Wagner et al. [1] by one trained moderator (postdoctoral research fellow with extensive experience in the dairy industry). Consent forms were signed prior to participation in the focus group discussion, and participants received an incentive ($100 Visa gift card) by mail following completion of the conference. The standard questions posed to focus groups’ participants are provided in Table 1. Focus group discussions were audio-recorded and transcribed verbatim by one trained, research team member.

#### 2.2.3. Qualitative Focus Group Data Analysis

Transcribed focus group discussions were systematically analyzed by two independent coders in accordance with methods described by Wagner et al. [1]. Coder 1 is a public health veterinarian and a PhD level researcher with experience and expertise in statistical mixed methods approaches with an emphasis on qualitative data analysis. Coder 2 is a postdoctoral research scholar in animal welfare with a PhD and expertise in cattle health and physiology. Focus group discussion analysis was quantified and results were presented as a percentage of the total discussion.

## 3. Results

### 3.1. Part I: Demographics

A total of 124 responses were recorded. Sixty-three respondents were removed based on false-duplicate IP addresses identified, or if they only responded to the demographics questions. These screening criteria resulted in sixty-one surveys qualifying for analysis. Regarding gender, 48% of respondents identified as males, 52% as females. Survey respondent’s median age was 37 years (25 to 73 years). The median number of years of experience as a dairy cattle veterinarian was 12 years (0.5 to 49 years). Respondents serviced a median of 12 dairy facilities and this number was not different when comparing veterinarians with <10 years of experience to those with >10 years of experience.

The median number of adult cows on serviced facilities was 625 (60 to 24,000). The median number of heifers and calves was 363 (16 to 50,000). All respondents worked with mature cows as well as weaned heifers (93.4% worked with weaned heifers) and pre-weaned calves (95.1% worked with pre-weaned calves), while only 45.9% of respondents indicated that they worked with bulls.

Only 13.1% of respondents were the primary decision maker regarding euthanasia, and 44.2% indicated that the person primarily responsible for making euthanasia decisions was dependent upon the facility. An equal proportion of respondents indicated that the farm owner (34.6%) or farm manager (34.6%) were primarily responsible for making on-farm euthanasia decisions.

In the past 12 months, 81.9% of respondents indicated that they had euthanized dairy cattle and 77.0% of euthanized cattle were adult cows. Only 3.3% of respondents perform the majority of euthanasia on the dairy facilities where they serve as the veterinarian, while 68.9% indicated that someone else was primarily responsible for performing euthanasia, and 27.9% indicated that this was farm dependent. A similar proportion of respondents indicated that the farm owner (28.5%) and farm manager (30.9%) were primarily responsible for performing on-farm euthanasia.

The majority of respondents (60%) indicated that most facilities for which they serve as the primary veterinarian have a written euthanasia protocol in place, and 94.5% of respondents were involved in the development of these protocols as the servicing veterinarian. The majority of respondents indicated that they were “only sometimes” consulted on individual euthanasia cases (67.2%), and 6.6% of respondents indicated that they are never consulted.

### 3.2. Part I: Decision Making about Health Conditions

Questions and respondent responses regarding the management of specific health conditions for each production stage are provided in Table 2. Regardless of the production stage, most respondents indicated that they would treat and monitor for all health conditions considered in the present study except for three conditions. In adult cattle, most respondents elected to cull/sell for beef for Johne’s and Lymphoma (93.3% and 50.8%, respectively) and euthanize non-ambulatory cattle regardless of production stage (54.1%, 58.7%, and 53.8% for adult cows, weaned heifers, and pre-weaned calves).

### 3.3. Part II: Focus Group

#### Participation and Outcomes

Twenty-two dairy veterinarians participated in one of three focus groups (9, 4, or 9 veterinarians/group, respectively). Questions about euthanasia were posed by a moderator and three main themes (logistical, animal, and human factors) and 16 subthemes were identified (Table 3) via systematic focus group discussion analysis. The majority of the discussion focused on logistical factors (37% of the discussion), followed by animal factors (33%) and human factors (30%).

Logistical Factors included seven subthemes and represented the largest proportion of focus group discussions. Logistical factors were named as such to encompass any discussion pertaining to protocols, materials needed, and financial considerations affecting on-farm euthanasia. Financial considerations, both personal (i.e., as the veterinarian being paid by the farm owner) and in relation to farm economics (i.e., return on investment for that animal), represented a significant portion of discussion within this main theme. In addition, the purpose and usefulness of euthanasia protocols were explored. Some participants noted the foundational purpose of implementing on-farm euthanasia protocols, stating, “Help improve employee compliance and recognition of the need to do this when it needs to be done.” Many other focus group participants frequently expressed frustration with protocol limitations and requirements, emphasizing a desire for more interactive training opportunities (between themselves and producers).

Animal Factors, comprised of five subthemes, encompassed any discussion that focused on the subjective experience (i.e., affective state) of the individual animal or animals on the farm. Animal welfare and animal status or condition were the most discussed subthemes, accounting for 73% of all Animal Factor discussion and 24% of the total focus group conversation. For example, in response to Q1 (What comes to mind when you think about euthanizing animals on-farm?), one participant touched on three subthemes (“animal welfare”, “health status” and “improvement”), stating “Certainly welfare…Prognosis is a big one. Trying to weigh the odds…what are the chances of the survival.”

Lastly, human factors were named as such to encompass any aspects of the discussion that focused on human considerations and included topics ranging from personal or perceived feelings, to human safety and public/client perception. For example, one participant expressed feelings of concern regarding client perception and stated, “She’s got to be put down now but you still have to consider client relations…do I really want to piss you off enough that I’m not going to come back again or lose money or can’t take care of the rest of the animals.” Others discussed the importance of training farm staff on appropriate methods of euthanasia as being critical to safeguarding human safety on-farm. Although human factors were discussed the least quantitatively, qualitative evaluation elucidated an undercurrent of the human emotion component, such as issues of morality or feelings of sadness, was present throughout focus group discussions.

## 4. Discussion

As experts in animal husbandry and disease, veterinarians serve as a valuable resource to animal producers. In the US dairy industry, veterinarians are relied upon to not only make recommendations regarding individual euthanasia cases [1] but also to develop comprehensive euthanasia protocols outlining if and when producers should euthanize a compromised animal [6,7].

Euthanasia is a readily-available tool used to decrease incidences of poor welfare when cattle are unlikely to recover [8,9]. This process involves the timely identification of compromised animals, appropriate treatment, on-going monitoring and implementation of euthanasia when animals fail to respond to treatment [6,7]. However, timely euthanasia remains a challenge for US dairy farms, and previous work by our group demonstrates inconsistencies amongst US dairy producers in regards to implementing timely euthanasia on-farm [1].

To minimize animal suffering for compromised cattle, the present study aimed to identify decision-making criteria that influence on-farm euthanasia of dairy cattle from a veterinarian’s perspective. The results of this study identified three main criteria that influence and may potentially impede a timely euthanasia response on-farm: (1) variability in health condition management amongst dairy veterinarians, (2) limited use of written euthanasia protocols on-farm, and (3) financial considerations and client relationships as influential factors when making euthanasia decisions.

### 4.1. Health Condition Management

In the present study, dairy cattle veterinarians varied regarding the management of health conditions and demonstrated a strong proclivity to provide treatment to compromised cattle. Of the 15 conditions described within this survey, veterinarians selected to treat all but three conditions including Johne’s, Lymphoma, and non-ambulatory. This outcome suggests that veterinarians regard treatment as the most valuable option for improving welfare by mitigating health challenges. Regardless of formal training or licensing requirements, veterinarians and producers demonstrated similar decision-making criteria regarding euthanasia [1]. Moreover, the majority of participating veterinarians selected to treat conditions for which recovery can be challenging (i.e., severely lame and non-ambulatory). It is possible that the simple presentation of health condition management questions in the survey (i.e., no background information was provided beyond the condition or disease name) contributed to the respondent’s disinclination to choose “euthanize immediately” given that numerous factors are considered when making euthanasia decisions in real-life scenarios. Nevertheless, the poor likelihood of recovery for some of the conditions considered [4,10,11,12] warrants additional attention. Future work should focus on providing veterinarians with the most up-to-date, science-based information to help define at what point treatment is no longer effective and euthanasia is necessary. Moreover, the development and dissemination of condition-specific decision tree models may provide further support for implementing evidence-based decision-making.

Health condition management for all production stages varied amongst veterinarians participating in the survey. For example, for adult cows with nervous system disorders, 29.5% of veterinarians elected to euthanize immediately, while 67.2% elected to treat and monitor, and 3.3% elected to cull/sell for beef. In addition, for weaned heifers exhibiting severe lameness, 23.9% of veterinarians would euthanize immediately, followed by 60.9% to treat and monitor, and 15.2% to cull/sell for beef. This is likely due to condition severity variability, differences in professional experience and specialization, as well as distinctive regional opportunities and potential constraints impacting dairy operations. Therefore, euthanasia cases are not always clear-cut and future work needs to focus on developing timelines specific to conditions with variable responses to treatment outcome.

### 4.2. Written Euthanasia Protocols

Results from the survey elucidated that >40% (estimated based on survey respondent perceptions) of facilities may not have a written euthanasia protocol in place, and over 90% of these protocols were written and developed by the veterinarian. Standard operating procedures are often overlooked but are critical documents used on-farm to provide guidance for specific animal management practices and procedures [13]. Written protocols are particularly important for euthanasia as they often include appropriate techniques to ensure the animal is rendered unconscious and includes information pertinent to timelines, confirmation of death, and human safety [8].

However, results from this study suggest that 40% of farms surveyed are without a protocol. Therefore, as an initial first step in improving timely euthanasia on-farm, veterinarians should ensure a euthanasia protocol is not only written, but visibly available to caretakers and provided in the native language of those that work with cattle. This is also important given that written protocols on euthanasia are often required by certification programs such as the National Milk Producers Farmers Assuring Responsible Management (FARM) Program^®^ [5] and Validus^®^ Dairy audit standards [14]. The FARM program^®^ encourages veterinarians to assist in preparing protocols and requires evaluators and auditors to assess each farm to ensure such protocols are in place [5]. Providing access to such information may result in the development of a strong farm culture to utilize euthanasia as a tool to end suffering and enhance knowledge on appropriate euthanasia timelines and techniques amongst farm staff [15].

However, writing and posting a protocol is only half the battle. Focus group participants frequently expressed frustration with protocol limitations and requirements, emphasizing that the highly interactive and variable nature of the euthanasia process limits the value of written protocols. Likewise, given veterinarians are often tasked with the job to write the protocol, protocols may be written with language that is misinterpreted, thus limiting comprehension of the protocols for producers and caretakers. One focus group participant called attention to these challenges, stating, “…we’ve given them a lot of responsibility…The worst part is they don’t ever go to look at them again…This is very much an interactive type learning process at the animal, otherwise it doesn’t stick very long.” Another participant voiced frustration regarding the limited interactive training with producers and stated, “We can’t get in there to do the training because they don’t want to spend the time or money on you...we’re forced into these protocols which make us uncomfortable at night.”

Given these frustrations, veterinarians must rely on a multimodal approach to train producers on euthanasia and may only be successful if written protocols are accompanied by training workshops with interactive components. This approach allows the veterinarian to develop a formal protocol on euthanasia but also work directly with the producer and caretakers to train them more effectively. This approach has been successful in the US swine industry, as work conducted by Campler and colleagues demonstrated that an interactive euthanasia training program improved caretaker perception of euthanasia decision-making knowledge when written protocols were previously established [16].

Veterinarians can also utilize decision-trees to promote appropriate decisions regarding euthanasia. Decision trees are an extremely useful tool in a wide variety of disciplines and practical applications because they are relatively simple [17], efficient, and provide support to those making decisions [18]. By implementing a stronger training program and ensuring written protocols are available and accessible to all that work on-farm, veterinarians can play a larger role in improving animal welfare by ensuring timely euthanasia.

### 4.3. Logistical Factors

The present study identified that participating dairy cattle veterinarians discussed logistical factors more frequently than either animal factors or human factors, and more frequently than dairy producers when asked the same questions [1].

One-tenth of all focus group discussions focused on financial considerations, however, the context of this discussion point varied between personal and farm-driven decision-making. From a personal perspective, veterinarians must weigh the advantages and disadvantages of accommodating the needs of their clients while maintaining professional integrity. For example, one participant stated, “You may have very, very strong feelings about this [situation] but the affect you’ll have by forcing the situation is going be detrimental to your future interaction.” Such professional and moral quandaries can elicit various emotional reactions [19,20], with one participant even stating “I think it’s an uncomfortable question…usually implies that I got talked out of something I know I should have done.” In 2018, Dr. Meijboom reported on the moral plurality often faced by veterinarians working in animal production industries and suggested that accommodating the needs and wants of the client may result in the most ideal outcome for both parties [21]. Given that veterinarians are ethically obligated to prevent and mitigate suffering in animals, as demonstrated by the Veterinarian’s oath (“Being admitted to the profession of veterinary medicine, I solemnly swear to use my scientific knowledge and skills for the benefit of society through the protection of animal health and welfare, the prevention and relief of animal suffering, the conservation of animal resources, the promotion of public health, and the advancement of medical knowledge…”) [22], they likely experience dichotomic challenges associated with this suggestion to accommodate producer needs. Furthermore, accommodating client needs may be contributing to inconsistencies in health condition management identified in the present study. Supporting veterinarians by directly addressing this issue of moral plurality, either in their core curriculum or in secondary training opportunities, may have benefits beyond reducing variability such as improved emotional states. However, additional research is needed in this area to identify effective strategies for providing such support.

From a farm economics perspective, treatment costs and cull eligibility directly influenced euthanasia decisions but were often dependent on farm size. The size of the operation or farm directly influenced veterinarian perception in regards to the weight that economics contributes to the decision-making process, client ability to provide supportive care, and the overall difficulty of carrying out euthanasia on-farm (e.g., equipment, carcass disposal). In general, larger farms were observed to be more likely to consider economics but also better equipped to provide supportive care and perform timely euthanasia.

## 5. Conclusions

In conclusion, veterinarians servicing the US dairy industry are heavily relied upon to provide guidance regarding on-farm euthanasia. However, variability regarding health condition management was identified amongst dairy cattle veterinarians in the present study, mirroring previously reported trends amongst dairy producers. Focus group discussion emphasized financial and client-relation factors impacting on euthanasia timeline decisions. In addition, participants frequently expressed frustration with limitations associated with written euthanasia protocols. Ensuring that all individuals involved in the on-farm euthanasia process have access to science-based standards and the development of interactive training programs remains a critical next step to improving cattle welfare consistency across the United States dairy industry.

This study calls attention to some challenges perceived by veterinarians in regard to ensuring timely euthanasia for severely compromised dairy cattle. However, the results of the present study may be biased, given that veterinarians who participated in focus group discussion may represent a more proactive subpopulation of dairy cattle veterinarians. Additionally, the modest number of survey respondents remains a limitation of the study. Future work is needed to recruit a larger subpopulation of veterinarians to elucidate all perceived challenges currently impacting on-farm euthanasia decision making.

## Figures and Tables

**Table 1:** Questions utilized in a focus group discussion on timely euthanasia on dairy farms in the US.

Q#	Question
Q1	What comes to mind when you think about euthanizing animals on-farm?
Q2	What, if any, are the benefits of euthanizing animals on-farm?
Q3	What, if any, are the drawbacks to euthanizing animals on-farm?
Q4	When do you know it is the right time to euthanize an animal?
Q5	When do you know it is NOT the right time to euthanize an animal?
Q6	What are the main reasons why you would delay euthanasia?
Q7	What are the main reasons why you would NOT perform euthanasia?
Q8	What other factors might you consider when making the decision to euthanize animals on-farm?

**Table 2:** Response percentages of surveyed dairy cattle veterinarians (*n* = 48) when asked to select how they would manage the following conditions.

Condition ^1^	Euthanize Immediately (%)	Treat and Monitor for Signs of Improvement (%)	Cull/Sell for Beef (%)	Not Applicable (%)
Adult Cow (*n* = 60)				
Bloat	1.67	95.0	-	3.33
Cancer eye	15.0	40.0	33.3	11.7
Calving complications ^1^	1.69	94.9	3.39	-
Diarrhea ^2^	-	98.3	-	1.67
Johne’s disease	-	5.00	93.3	1.67
Ketosis/Milk fever	-	100	-	-
Lameness, severe ^3^	11.7	61.7	26.7	-
Lymphoma ^4^	41.0	1.64	50.8	6.56
Nervous system disorders ^5^	29.5	67.2	3.28	-
Non-ambulatory/Downer	54.1	45.9	-	-
Pneumonia	-	96.7	1.64	1.64
Toxic mastitis	-	95.1	4.92	-
Traumatic injury	27.9	72.1	-	-
**Weaned Heifers** (*n* = 46)				
Bloat	-	97.8	2.17	-
Diarrhea	-	95.7	4.35	-
Joint infection	13.0	65.2	19.6	2.17
Lameness, severe	23.9	60.9	15.2	-
Navel/Umbilical infection	-	100	-	-
Nervous system disorder	17.4	82.6	-	-
Non-ambulatory/Downer	58.7	41.3	-	-
Pneumonia	-	100	-	-
Traumatic injury	34.8	63.0	-	2.17
**Pre-weaned Calves** (*n* = 39)				
Bloat	2.56	97.4	-	-
Diarrhea	-	97.5	-	2.50
Joint infection	2.56	94.9	2.56	-
Lameness, severe	20.5	79.5	-	-
Navel/umbilical infection	-	100	-	-
Nervous system disorder	18.0	82.0	-	-
Non-ambulatory/Downer	53.8	46.2	-	-
Pneumonia	-	97.4	2.56	-
Traumatic injury	29.0	71.0	-	-

^1^ Paralysis, dystocia, prolapsed uterus, C-section. ^2^ Severe, with dehydration. ^3^ Severe; score of 3 on 3-point scale; score 4 on 4-point scale; score 5 on 5-point scale. ^4^ Bovine leukosis. ^5^ Circling or incoordination; convulsions; involuntary eye movement; head tilt.

**Table 3:** The main themes and subthemes discussed by focus group participants, the proportion of time dedicated to each theme, and a brief, direct quote that highlights each theme.

Themes	% of Discussion	Direct Quotes
**Logistical Factors**		
Financial/economical	10	“The right time sometimes becomes an economic decision…in lieu of spending money, what are you going to do…”
Protocols/procedures/guidelines	8.9	“…remove the discussion from being in the heat of the moment…have it in advance. So, I do think there is value in protocols and SOPs and decision trees.”
Client/operation/farm size	6.5	“…not so much our larger clients but for a lot of smaller clients, they, I think, have reservations…”
Carcass disposal	4.5	“Depending on methodology, disposal of the carcass can be difficult. If the vet has to use barbiturates…then they have to bury…or incinerate, which is cost prohibitive.”
Time/labor/space	3.1	“…from the allocation of labor resources [standpoint]…down cows are really time consuming, and I think it’s important to make that decision early.”
Equipment	2.4	“With the use of captive bolt you need to be very aware of what your secondary kill step is gonna be. If that’s gonna be a second bolt…if that’s gonna be KCl IV.”
Ownership	1.7	“When the owner is not ready, unfortunately…legally it’s not our right to make the decision.”
**Animal Factors**		
Health status/condition/disease	12	“If you have an animal…diagnosed with an irreversible disease…like for instance Johne’s disease is going to be spreading disease everywhere…those are things we definitely…use as criteria to euthanize.”
Animal welfare/wellbeing	12	“…[euthanasia] can improve welfare and does improve welfare, particularly so we’re not having to move those animals.”
Improvement	5.3	“It’s a strong judgement issue, but to actually see an animal progress…and how far they’ve improved…”
Slaughter eligibility	1.9	“Some cows that are not fit for transport go to slaughter anyway. So, euthanasia at the farm, to me, helps with welfare of these animals.”
Herd impact	1.7	“If her last thing she can do to contribute to the herd is to find out why…did she get that way...that may be her lasting benefit—you didn’t make any money…but [she] contributes to that not happening again.”
**Human Factors**		
Emotions/psychology	14	“…with that comes the compassion fatigue and emotional stress and we see that there is benefit in dissociating…”
Education/training	7.2	“...training comes into it from a decision-making perspective, looking at making that decision timely.”
Human safety/food safety	6.0	“…we know it’s not the right time to euthanize if it can’t be done humanely and safely…for safety of humans.”
Public/client perception	3.1	“…who is going to see [animals down and sick and visibly in discomfort] and who is then going to potentially share it with others and make that seem like that’s a norm when I don’t think it necessarily is.”

## Appendix A

**Table A1:** Survey questions used to attain respondent demographic information, including the response options provided for each question.

Q#	Question Text	Response Options
2.1	What is your age?	*Text box provided*
2.2	What is your gender?	Male Female Other Prefer not to answer
2.3	Approximately how long have you worked with dairy cattle as a veterinarian?	*Text box provided*
2.4	How many dairy facilities do you serve as the primary veterinarian?	*Text box provided*
2.5	Please provide the range of the number of adult cows (lactating and dry) on the dairy facilities where you serve as a veterinarian.	*Text box provided*
2.6	Please provide the range of the number of heifers and calves on the dairy facilities where you serve as a veterinarian.	*Text box provided*
2.7	With which of the following cattle groups do you currently work? (select all that apply)	Pre-weaned calves Weaned heifers Mature cows Bulls
2.8	In the past 12 months, have you euthanized any dairy cattle?	Yes No
	If yes → Q2.9: In the past 12 months, which groups of dairy cattle have you euthanized? (select all that apply)	Dairy bulls or dairy yearling bulls Dairy steers/beef Pre-weaned calves (calves still on milk) Weaned heifers Adult cows
2.10	Do you perform the majority of euthanasia on the dairy facility where you serve as the veterinarian?	I do Someone else It depends on the facility, please explain: *Text box provided*
	If someone else → Q2. 11: If someone else performs most euthanasia, what is this person’s role?	Farm owner Farm manager Animal caretaker/employee Renderer Other, please specify: *Text box provided*
2.12	On most dairy facilities where you serve as veterinarian, who makes the decision to euthanize an animal?	I do Someone else It depends on the facility, please explain: *Text box provided*
	If someone else → Q2.13: If someone else makes the decision to euthanize, what is this person’s role?	Farm owner Farm manager Animal caretaker/employee It depends on the facility, please explain: *Text box provided*
2.14	Do most dairy facilities where you serve as the veterinarian have a written protocol for dairy cattle euthanasia?	Yes No
2.15	In your role as the facility’s veterinarian, were you involved in making the euthanasia protocol?	Yes No
2.16	In the past 12 months, how often did your clients consult you before euthanizing dairy cattle?	Always/every case Often/most cases Sometimes/a few cases Never/no cases
